# ARGET-ATRP-Mediated Grafting of Bifunctional Polymers onto Silica Nanoparticles Fillers for Boosting the Performance of High-Capacity All-Solid-State Lithium–Sulfur Batteries with Polymer Solid Electrolytes

**DOI:** 10.3390/polym16081128

**Published:** 2024-04-17

**Authors:** Liang Wang, Junyue Huang, Yujian Shen, Mengqi Ma, Wenhong Ruan, Mingqiu Zhang

**Affiliations:** 1 Key Laboratory for Polymeric Composite and Functional Materials of Ministry of Education, GD HPPC Lab, School of Chemistry, Sun Yat-sen University, Guangzhou 510275, China; wangln57@mail2.sysu.edu.cn (L.W.); huangjy329@mail2.sysu.edu.cn (J.H.); shenyj7@mail2.sysu.edu.cn (Y.S.); mamq6@mail2.sysu.edu.cn (M.M.); ceszmq@mail.sysu.edu.cn (M.Z.); 2 Guangdong Provincial Laboratory of Chemistry and Fine Chemical Engineering Jieyang Center, Jieyang 515200, China

**Keywords:** ARGET-ATRP, silica nanoparticles, lithium–sulfur batteries, polymer solid electrolytes

## Abstract

The shuttle effect in lithium–sulfur batteries, which leads to rapid capacity decay, can be effectively suppressed by solid polymer electrolytes. However, the lithium-ion conductivity of polyethylene oxide-based solid electrolytes is relatively low, resulting in low reversible capacity and poor cycling stability of the batteries. In this study, we employed the activator generated through electron transfer atom transfer radical polymerization to graft modify the surface of silica nanoparticles with a bifunctional monomer, 2-acrylamide-2-methylpropanesulfonate, which possesses sulfonic acid groups with low dissociation energy for facilitating Li^+^ migration and transfer, as well as amide groups capable of forming hydrogen bonds with polyethylene oxide chains. Subsequently, the modified nanoparticles were blended with polyethylene oxide to prepare a solid polymer electrolyte with low crystallinity and high ion conductivity. The resulting electrolyte demonstrated excellent and stable electrochemical performance, with a discharge-specific capacity maintained at 875.2 mAh g^−1^ after 200 cycles.

## 1. Introduction

With the continuous growth in global energy demand, lithium-ion batteries (LIBs) with high energy density and long cycling capability have experienced widespread development, playing an increasingly significant role in the global energy system [1,2,3]. The remarkable specific capacity (1672 mAh g^−1^) and cost-effectiveness have garnered extensive attention for sulfur cathodes in the field of lithium batteries [4]. Among them, lithium–sulfur (Li-S) batteries, which are compatible with the coveted lithium metal as the anode, represent one of the most extensively researched types of LIBs [5,6,7,8].

However, in conventional liquid-state LIB systems, the favorable solubility of lithium polysulfides (LiPSs) generated at the cathode during battery cycling leads to continuous loss of active materials in the cathode [8,9,10,11,12]. These soluble LiPSs (Li_2_S_x_, 4 < x < 8) migrate to the anode side with the electrolyte, a phenomenon known as the “shuttle effect”, where they react with the lithium metal, forming insoluble Li_2_S_2_ and Li_2_S, which subsequently deposit on the lithium metal surface [13,14,15]. This seemingly promising cathode material simultaneously gives rise to the aforementioned issues on both the cathode and anode, resulting in rapid capacity decay of Li-S batteries. The development of all-solid-state Li-S batteries holds tremendous potential for the commercialization of Li-S battery technology [16,17,18,19,20]. Solid polymer electrolytes (SPEs) can play a crucial role by acting as physical barriers, effectively controlling the dissolution of LiPSs from the cathode and mitigating their negative impact on the anode [18,21,22,23,24]. Additionally, SPEs offer a range of advantages, such as preventing electrolyte leakage, enabling high energy density, and enhancing safety [24,25,26]. In the realm of SPEs, blends of high molecular weight polyethylene oxide (PEO) and its derivatives with lithium salts have gained considerable attention [27,28,29,30,31]. This stems from the favorable attributes of PEO, including excellent mechanical properties, high solubility with lithium salts, and favorable chemical stability towards lithium metal [32]. The mechanism by which PEO-based polymer solid electrolytes facilitate Li^+^ transport is through the coordination of Li^+^ ions, dissociated from lithium salts, with ether oxygen atoms along the PEO polymer chains. The migration and transfer of Li^+^ ions are driven by the segmental motion of the PEO polymer chains [33,34]. However, it should be noted that PEO belongs to the category of crystalline polymers, exhibiting a relatively high degree of crystallinity at lower temperatures. This characteristic hinders the effective conduction of Li^+^ ions through the crystalline regions of the PEO polymer chains, resulting in low lithium-ion conductivity (approximately 10^−7^ S cm^−1^) [31]. Consequently, batteries utilizing PEO-based solid electrolytes may exhibit limited reversible capacity and suboptimal cycling stability [35,36,37].

The incorporation of diverse nanostructured inorganic fillers with varying surface properties into the polymer matrix of PEO to form composite SPEs represents a promising avenue to mitigate its crystallinity and enhance the performance of SPEs [38,39,40]. For instance, the crystallinity of PEO could be reduced by introducing Al_2_O_3_ or SiO_2_ fillers into the polymer matrix [41,42,43,44,45,46]. This approach effectively increased the number of free polymer segments, thereby accelerating the segmental motion of the polymer chains and ultimately leading to enhanced lithium-ion conductivity.

In our previous investigations, we observed the tendency of inorganic fillers with abundant polar functional groups to aggregate when incorporated into crystalline polymer matrices for modification [47]. Such poor compatibility hampers the efficiency of filler-mediated polymer modification, particularly when the filler itself is inert and lacks inherent Li^+^ conductivity. To address this challenge, surface modification of inorganic fillers has emerged as a viable approach. Motivated by this concept, we employed cost-effective and readily available silica nanoparticles (SiO_2_ Nps), a typical inert filler, and employed the activators regenerated by the electron transfer atom transfer radical polymerization (ARGET-ATRP) technique to graft a bifunctional monomer lithium 2-acrylamide-2-methylpropanesulfonate (AMPSLi) onto the filler surface (SiO_2_-g-PAMPSLi). This monomer possesses sulfonic acid moieties with low dissociation energy for facilitating Li^+^ migration and transfer, as well as amide groups capable of forming hydrogen bonds with PEO polymer chains. Subsequently, the modified filler was blended with PEO to fabricate SPEs with reduced crystallinity and enhanced ion conductivity.

## 2. Materials and Methods

### 2.1. Preparation of SiO_2_-g-PAMPSLi

**Figure 1:**
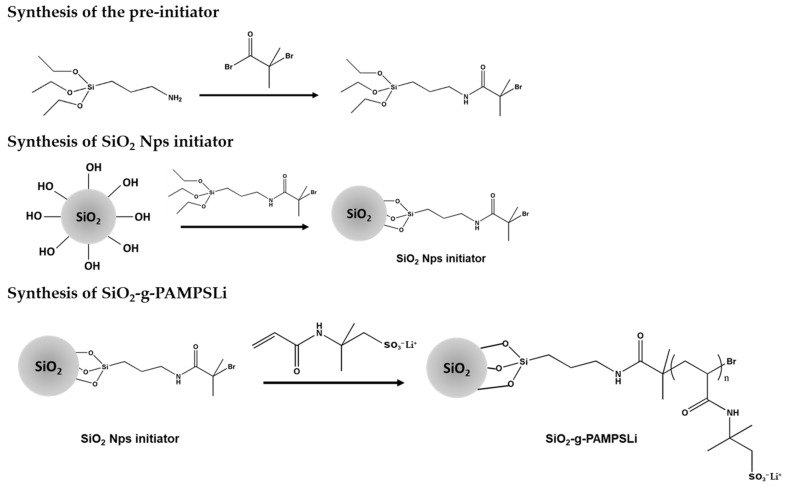
Preparation of SiO_2_-g-PAMPSLi.

Firstly, SiO_2_ NPs were obtained by using a modified Stöber method and activated by hydrogen peroxide and concentrated sulfuric acid [48]. Then, a certain amount of 2-bromo-2-methylpropanoyl bromide, 3-aminopropyltriethoxysilane, and triethylamine were mixed to produce the siloxane pre-initiator. Under anhydrous and anaerobic conditions, SiO_2_ NPs and siloxane pre-initiator reactions formed the SiO_2_ Nps initiator. Then, with the initiator and AMPSLi added, the SiO_2_-g-PAMPSLi nanoparticle was successfully obtained through ARGET-ATRP. The concrete process of synthesis is shown in the Supplementary Materials as well as Figure 1.

### 2.2. Preparation of SiO_2_-g-PAMPSLi@PEO-Based Polymer Composite Solid Electrolytes

This experiment uses a solution pouring method to prepare solid electrolytes, with the specific steps as follows: (1) dissolve a certain amount of LiClO_4_ in 10 mL acetonitrile according to the EO/Li^+^ ratio [49]; (2) add a certain amount of inorganic solid powder to the above solution and sonicate for 15 min to disperse; (3) add 0.88 g of polyethylene oxide (PEO) and a certain amount of SiO_2_-g-PAMPSLi to the above dispersion and stir at room temperature for 24 h; then (4) pour the solution into a PTFE mold with length, width, and height of 50 mm × 50 mm × 3 mm. First, let the solvent evaporate naturally under a fume hood. Then, remove the solvent on a 40 °C hot bench and dry it in a 40 °C vacuum oven for 24 h. (5) Lastly, cut the dried solid electrolyte film into circular slices with a diameter of 16 mm using a slicer for later use.

### 2.3. Preparation of Carbon Sulfur Composite Cathode Materials

This experiment used an in-situ loading method to load sulfur onto Super P-activated carbon to prepare active materials. The specific steps are as follows: (1) dissolve and disperse 3 g sodium thiosulfate pentahydrate and 0.15 g Super P-activated carbon in 10 mL deionized water, stir for 15 min; (2) dissolve 2.37 g of concentrated hydrochloric acid in 23.7 mL of deionized water and slowly add it to the above solution through a constant pressure drip funnel under magnetic stirring; (3) react at room temperature for 12 h; (4) solid and liquid are separated by suction filtration, and the filter residue is washed with deionized water until the filtrate is neutral. It is then dried in a vacuum oven at 60 °C for 24 h before being set aside.

### 2.4. Preparation of Cathodes and Lithium–Sulfur Batteries

This experiment uses the scraping coating method to prepare the cathode of lithium–sulfur batteries. The specific steps are as follows: (1) grind and mix 0.8 g of carbon–sulfur composite material, 0.1 g of acetylene black, and 0.1 g of polyvinylidene fluoride (in a mass ratio of 8:1:1) evenly in an agate mortar; (2) add the mixed powder to 5 mL of N-methylpyrrolidone and stir at room temperature for 12 h to obtain a viscous cathode slurry; (3) use a four-sided scraper to coat the cathode slurry onto the carbon coated aluminum foil, and dry it in a 60 °C blast oven for 12 h to remove solvents; (4) cut the carbon-coated aluminum foil coated with cathode material and dried into circular pieces with a diameter of 12 mm using a slicer, and store them in a glove box filled with argon gas atmosphere for later use.

### 2.5. Electrochemical Measurements

The solid electrolytes, cathodes, and lithium metal were assembled into the CR2032 button battery in a glove box filled with argon gas. The electrochemical stability window was tested by linear sweep voltammetry (LSV) measurement under a scan rate of 5 mV s^−1^, from 2.0 to 6.0 V (vs. Li/Li+) in an electrochemical workstation (CHI760E, CH Instrument, Shanghai, China). The cyclic voltammetry (CV) was carried out at a scan rate of 0.1 mV s^−1^ between 1.6 and 2.8 V to characterize the redox reaction and reversibility. The electrochemical cycling performance of the coin cells was tested within a voltage window of 1.6–2.8 V using a battery test system (CT2001A, LAND, Wuhan, China).

## 3. Results

Characterization of the synthesized pre-initiator was performed by dissolving it in deuterated chloroform and analyzing its chemical structure using nuclear magnetic resonance (NMR) spectroscopy. Figure S1 presents the proton NMR spectrum of the pre-initiator. From the spectrum, it can be observed that the absorption peaks at 1.21 ppm and 3.97 ppm correspond to the methyl and methylene protons on the ethoxy groups, respectively. The peaks at 0.63 ppm, 1.61 ppm, and 3.26 ppm correspond to the three methylene protons on the original amino propyl group. The peak at 6.89 ppm corresponds to the hydrogen atom in the amide group of the pre-initiator, while the peak at 1.98 ppm corresponds to the two methyl protons on the α-carbon atom connected to the carbonyl group. The ratios of the peak areas of all characteristic peaks are in complete agreement with the proportions of hydrogen atoms in the chemical structure, indicating the successful synthesis of the pre-initiator.

**Figure 2:**
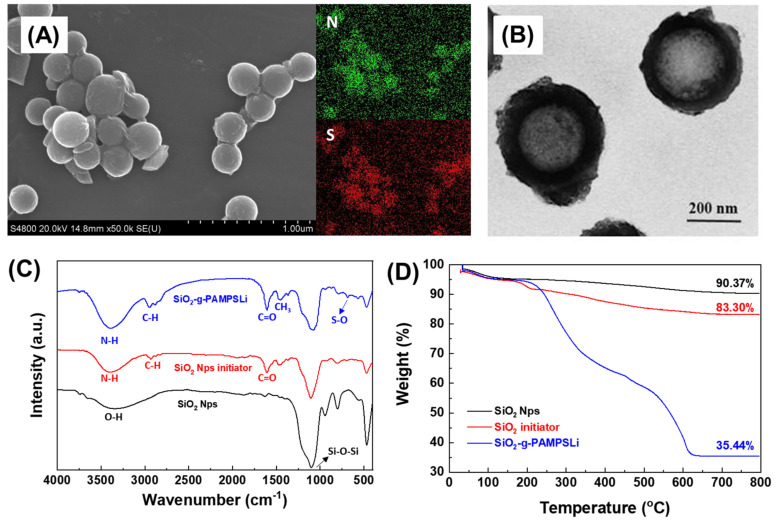
(**A**) SEM and corresponding EDS element scanning profiles and (**B**) TEM images of SiO_2_-g-PAMPSLi; (**C**) infrared spectra and (**D**) thermogravimetric curves of SiO_2_ Nps, SiO_2_ Nps initiator, and SiO_2_-g-PAMPSLi.

In order to investigate the morphological structure of SiO_2_-g-PAMPSLi, scanning electron microscopy (SEM) and transmission electron microscopy (TEM) were employed for characterization. As shown in Figure S2, the surface of SiO_2_ Nps particles appears smooth and uniform. However, in Figure S3, the SiO_2_ Nps initiator exhibits the presence of adhered substances, indicating the successful grafting of the pre-initiator onto the surface of SiO_2_ Nps. After the ARGET-ATRP polymerization reaction, Figure 2A clearly reveals that the surface of SiO_2_ particles is no longer smooth, exhibiting noticeable adhered substances and agglomeration between particles. This phenomenon suggests the successful polymerization of AMPSLi monomers on the surface of SiO_2_ particles, forming a polymer layer that encapsulates the SiO_2_ particles and leads to interparticle adhesion. Elemental mapping analysis using energy-dispersive X-ray spectroscopy (EDS) confirms the presence of nitrogen (N) and sulfur (S) elements on the surface of SiO_2_-g-PAMPSLi particles, which are exclusive to the AMPSLi monomers. This observation further confirms the successful surface modification of SiO_2_ Nps. Moreover, the TEM image in Figure 2B reveals that SiO_2_-g-PAMPSLi nanoparticles possess a core–shell structure, with the SiO_2_ core and a PAMPSLi polymer shell formed by the polymerization of AMPSLi monomers. Infrared spectroscopy was performed on SiO_2_ Nps, SiO_2_ Nps initiator, and SiO_2_-g-PAMPSLi, as shown in Figure 2C. For SiO_2_ Nps, the strong absorption peak at 1100 cm^−1^ corresponds to the stretching vibration of Si-O-Si bonds, while the peak at 3300 cm^−1^ corresponds to the stretching vibration of hydroxyl groups (O-H) on the surface of SiO_2_ Nps. For the SiO_2_ Nps initiator, the peak at 3400 cm^−1^ corresponds to the stretching vibration of N-H bonds on the amine group, the peak at 2925 cm^−1^ corresponds to the stretching vibration of saturated C-H bonds, and the peak at 1620 cm^−1^ corresponds to the stretching vibration of C=O double bonds on the carbonyl group. These three characteristic peaks correspond to the functional groups present in the pre-initiator, and the peaks at 1465 cm^−1^ and 1402 cm^−1^ correspond to the antisymmetric and symmetric deformation vibrations of the methyl groups on the pre-initiator, respectively, indicating the successful grafting of the pre-initiator onto the surface of SiO_2_ Nps to form the SiO_2_ Nps initiator. For SiO_2_-g-PAMPSLi, the monomer AMPSLi contains carbonyl, amine, and methyl groups. From the infrared spectrum, it can be observed that the intensities of the characteristic peaks corresponding to these three groups are enhanced. Additionally, the absorption peaks around 1070 cm^−1^ and 1193 cm^−1^ (overlapping with the stretching vibration peak of Si-O-Si bonds) as well as the peak at 630 cm^−1^ correspond to the antisymmetric and symmetric deformation vibrations and stretching vibration of S-O bonds on the sulfonic acid group, respectively. This further confirms the successful polymerization and grafting of AMPSLi monomers on the surface of SiO_2_ Nps, forming SiO_2_-g-PAMPSLi. Figure 2D presents the thermogravimetric analysis (TGA) curves of SiO_2_ Nps, the SiO_2_ Nps initiator, and SiO_2_-g-PAMPSLi. A notable observation is the 7% higher weight loss in the SiO_2_ Nps initiator compared to SiO_2_ Nps, confirming the successful bonding of the organic component to the surface of SiO_2_ Nps and the successful synthesis of the SiO_2_ initiator. Furthermore, a distinct weight loss is evident in SiO_2_-g-PAMPSLi beginning at 210 °C, indicative of the polymer grafting on the surface of SiO_2_ Nps. This finding allows for the calculation of the mass fraction of the grafted polymer PAMPSLi on the SiO_2_ Nps surface, estimated to be approximately 64.56%.

Composite SPEs were prepared by incorporating SiO_2_ Nps and SiO_2_-g-PAMPSLi into PEO (SiO_2_@PEO and SiO_2_-g-PAMPSLi@PEO). The morphology of the two composites was examined using SEM (Figure S3). In comparison to SiO_2_@PEO, SiO_2_-g-PAMPSLi@PEO exhibited a more uniform dispersion of fillers. Even at a high loading of 20 wt%, the SiO_2_-g-PAMPSLi particles did not aggregate. This can be attributed to the presence of the PAMPSLi polymer layer, which effectively isolates the inorganic SiO_2_ particles, preventing their agglomeration. Furthermore, the polymer layer demonstrates excellent organic–organic compatibility with the PEO matrix, facilitating the homogeneous dispersion of SiO_2_-g-PAMPSLi particles within the PEO matrix. However, at a loading of 25 wt%, SEM images revealed the occurrence of particle aggregation in SiO_2_-g-PAMPSLi@PEO.

**Figure 3:**
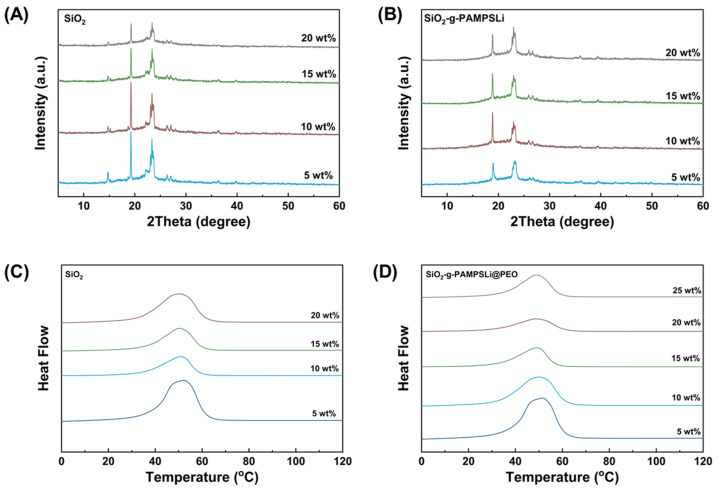
The XRD spectra and DSC curves of SPEs containing varying amounts of (**A**,**C**) SiO_2_ and (**B**,**D**) SiO_2_-g-PAMPSLi.

To investigate the influence of nanoparticles on the crystallization behavior of PEO, X-ray diffraction (XRD) and differential scanning calorimetry (DSC) characterization was performed on SPEs with different contents of SiO_2_ (Figure 3A,C) and SiO_2_-g-PAMPSLi (Figure 3B,D). The melting temperatures, enthalpies of fusion, and crystallinity of all substances in the DSC curves are summarized in Tables S1 and S2. The crystallinity of polymer PEO was calculated using the following equation.

$$\chi_{c}=\frac{\Delta H_{m}}{\Delta H_{PEO}\cdot f_{PEO}}\times 100\%$$

Here, $\chi_{c}$ represents the crystallinity of polymer PEO, and $\Delta H_{PEO}$ denotes the enthalpy of fusion for fully crystallized PEO, with a value of 196.4 J g^−1^. $\Delta H_{m}$ corresponds to the experimentally measured enthalpy of fusion, and $f_{PEO}$ represents the mass fraction of PEO in the SPEs.

The XRD spectra of individual SiO_2_ and SiO_2_-g-PAMPSLi exhibited no distinct diffraction peaks, indicating their amorphous nature. In comparison to the XRD spectrum of SiO_2_@PEO, the XRD spectrum of SiO_2_-g-PAMPSLi@PEO did not exhibit the diffraction peaks at 14.9° and 22.3°. This suggests the presence of interactions between certain functional groups on SiO_2_-g-PAMPSLi and the PEO chains, which may affect the crystallization behavior of PEO and disrupt the corresponding crystalline regions associated with these diffraction peaks. With an increase in the loading of SiO_2_-g-PAMPSLi, the intensity of the characteristic diffraction peaks of PEO at 19.2° and 23.5° decreased, indicating the ability of SiO_2_-g-PAMPSLi to reduce the crystallinity of PEO. The DSC curves reveal that although the melting temperature of SiO_2_-g-PAMPSLi@PEO shows a slight increase with increasing SiO_2_-g-PAMPSLi content, the intensity of the melting peak significantly decreases. This observation is consistent with the results of melting temperature and crystallinity presented in Table S1. As shown in Table S1, when the SiO_2_-g-PAMPSLi content is below 20 wt%, the crystallinity of SiO_2_-g-PAMPSLi@PEO notably decreases with increasing SiO_2_-g-PAMPSLi content, reaching a minimum value of 24% at 20 wt%. Nevertheless, when the SiO_2_-g-PAMPSLi content exceeds a certain threshold, particle aggregation occurs (Figure S3), resulting in reduced interaction with polymer PEO and increased crystallinity. Furthermore, compared to SiO_2_@PEO, SiO_2_-g-PAMPSLi@PEO exhibits lower crystallinity at the same filler content, and the difference becomes more pronounced as the filler content increases. This phenomenon can be attributed to the presence of abundant amine and unreacted hydroxyl groups on the surface of SiO_2_-g-PAMPSLi particles. The hydrogen atoms on these functional groups can form stronger hydrogen bonds with the ether oxygen atoms in PEO, surpassing the hydrogen bonding effect of the hydroxyl groups on SiO_2_. As a result, the impact on PEO crystallization is more pronounced, leading to a more effective reduction in PEO crystallinity. Additionally, the SiO_2_-g-PAMPSLi@PEO system achieves the lowest crystallinity at a higher filler content compared to SiO_2_@PEO. This is attributed to the excellent dispersion of SiO_2_-g-PAMPSLi particles in PEO, even at higher contents, as confirmed by the SEM image in Figure S3.

To investigate the interaction mechanism between SiO_2_-g-PAMPSLi particles and polymer PEO in SiO_2_-g-PAMPSLi@PEO, the mechanical properties were evaluated through tensile testing, as shown in Figure S4. PEO and SiO_2_@PEO with an equivalent filler content were selected as control groups. As observed from the graph, PEO exhibits a tensile strength of only 2.2 MPa and a fracture elongation of approximately 330% in the absence of filler addition. Upon the addition of nanoscale particle fillers, both SPE systems demonstrate a significant improvement in tensile strength and fracture elongation. This indicates that both types of nanoscale particles enhance and toughen the polymer PEO. Furthermore, SiO_2_-g-PAMPSLi exhibits superior reinforcement and toughening effects on polymer PEO compared to SiO_2_. This can be attributed to two factors: firstly, the surface of SiO_2_-g-PAMPSLi contains a substantial number of functional groups capable of interacting with PEO chains, thereby enhancing the tensile strength of SPEs. Secondly, at an equivalent filler content, SiO_2_-g-PAMPSLi@PEO exhibits lower crystallinity, leading to enhanced material toughness and increased fracture elongation. Figure S5 presents the stress–strain curves of SiO_2_-g-PAMPSLi@PEO with different SiO_2_-g-PAMPSLi contents. It can be observed that both the tensile strength and fracture elongation of SiO_2_-g-PAMPSLi@PEO increase with an increase in SiO_2_-g-PAMPSLi content. However, when the content reaches 25 wt%, the fracture elongation decreases. This behavior can be attributed to the stronger interaction between SiO_2_-g-PAMPSLi and polymer PEO at higher SiO_2_-g-PAMPSLi contents, leading to enhanced tensile strength and fracture elongation. Nevertheless, when the SiO_2_-g-PAMPSLi content exceeds a certain threshold, particle aggregation occurs, resulting in reduced interaction with polymer PEO, increased crystallinity, and decreased flexibility. Consequently, the fracture elongation decreases.

**Figure 4:**
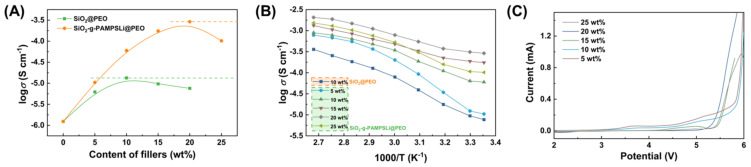
The relationship between lithium-ion conductivity of various SPEs and (**A**) filler loading levels, as well as (**B**) temperature; (**C**) Linear sweep voltammetry tests of SiO_2_-g-PAMPSLi@PEO composites with varying SiO_2_-g-PAMPSLi content.

The lithium-ion conductivity relationship with filler content at room temperature (25 °C) for SPEs is depicted in Figure 4A. It is evident that the lithium-ion conductivity of SiO_2_-g-PAMPSLi@PEO consistently surpasses that of SiO_2_@PEO at equivalent filler content. Moreover, as the filler content increases, the disparity between the two becomes more pronounced, reaching an order of magnitude higher lithium-ion conductivity for SiO_2_-g-PAMPSLi@PEO compared to SiO_2_@PEO at higher concentrations. On one hand, under equivalent content conditions, the crystallinity of SiO_2_-g-PAMPSLi@PEO is lower than that of SiO_2_@PEO, promoting an increase in the proportion of amorphous PEO molecular chains favorable for Li^+^ migration and transport, thereby enhancing lithium-ion conductivity. On the other hand, the surface of SiO_2_-g-PAMPSLi particles is rich in lithium sulfonate groups, facilitating interaction with Li^+^ to promote salt dissociation and possessing inherent Li^+^ conductivity. Consequently, SPEs incorporating SiO_2_-g-PAMPSLi exhibit significantly improved lithium-ion conductivity. However, when the SiO_2_-g-PAMPSLi content exceeds a certain threshold, the lithium-ion conductivity of SiO_2_-g-PAMPSLi@PEO decreases. This is attributed to the agglomeration of SiO_2_-g-PAMPSLi particles at higher concentrations, increasing the crystallinity of SiO_2_-g-PAMPSLi@PEO and consequently leading to a reduction in lithium-ion conductivity.

Temperature experiments on the lithium-ion conductivity of SiO_2_-g-PAMPSLi@PEO with varying SiO_2_-g-PAMPSLi content were conducted, using 10 wt% SiO_2_@PEO as a control group (Figure 4B). The results demonstrate that the lithium-ion conductivity of all SPEs increases with temperature elevation, particularly in the range of 40 °C to 70 °C. This is attributed to the transition of crystalline PEO chains to a molten state from 40 °C, enhancing the free movement of PEO chains and facilitating Li^+^ migration and transport, resulting in a noticeable increase in solid electrolyte lithium-ion conductivity. Beyond 80 °C, PEO enters a fully molten state, and the lithium-ion conductivity of solid electrolytes conforms to the Vogel–Tamman–Fuclcher equation with temperature variations [31].

Figure S5 presents the chronoamperometric curves and corresponding pre- and post-polarization AC impedance spectra at room temperature (25 °C) for SiO_2_-g-PAMPSLi@PEO with varying SiO_2_-g-PAMPSLi content, with the summarized test results provided in Table S3. It is observed that the lithium-ion transference number of SiO_2_-g-PAMPSLi@PEO increases with higher SiO_2_-g-PAMPSLi content. This enhancement can be attributed to the presence of the polymer PAMPSLi layer on the surface of SiO_2_-g-PAMPSLi particles, which not only facilitates Li^+^ migration and transport but also interacts with PEO molecular chains, reducing the crystallinity of PEO and promoting Li^+^ migration and transport.

Linear sweep voltammetry tests were conducted on SiO_2_-g-PAMPSLi@PEO composites with varying SiO_2_-g-PAMPSLi content, as depicted in Figure 4C. It is evident from the graph that with increasing SiO_2_-g-PAMPSLi content, the electrochemical stability window of SiO_2_-g-PAMPSLi@PEO also rises. Notably, all SiO_2_-g-PAMPSLi@PEO composites exhibit electrochemical stability windows exceeding 5.0 V (vs. Li/Li+), meeting the electrolyte requirements of Li-S batteries. Following a series of electrochemical performance assessments, it was determined that the electrochemical performance of the 20 wt% SiO_2_-g-PAMPSLi@PEO composite was optimal. Consequently, the 20 wt% SiO_2_-g-PAMPSLi@PEO composite was selected as the solid electrolyte for assembling solid-state Li-S batteries, which were subsequently subjected to further battery performance evaluations.

**Figure 5:**
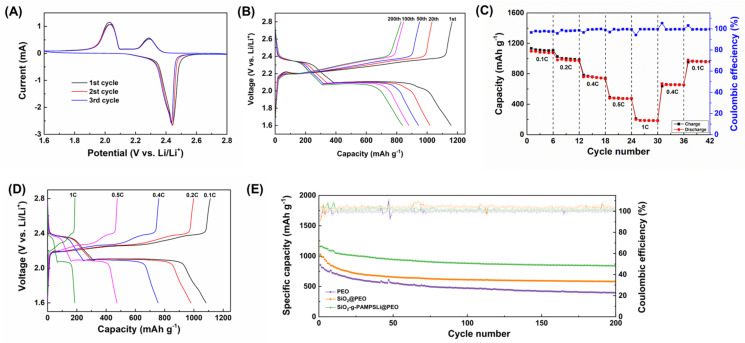
(**A**) Cyclic voltammetry curves of Li-S batteries employing SiO_2_-g-PAMPSLi@PEO as SPEs; (**B**) constant current charge–discharge profiles of Li-S batteries utilizing SiO_2_-g-PAMPSLi@PEO as SPEs under standard temperature (25 °C) and 0.1 C current rate conditions are also depicted; (**C**) rate performance and (**D**) charge–discharge profiles at current densities ranging from 0.1 C to 1 C of Li-S batteries employing SiO_2_-g-PAMPSLi@PEO as SPEs; (**E**) discharge capacities and Coulombic efficiencies of Li-S batteries employing PEO, SiO_2_@PEO, and SiO_2_-g-PAMPSLi@PEO as SPEs under standard temperature (25 °C) and 0.1 C current rate conditions.

Figure 5A illustrates the cyclic voltammetry curves of Li-S batteries employing SiO_2_-g-PAMPSLi@PEO as SPEs. The curves exhibit two distinct reduction peaks and one oxidation peak, indicative of the typical multi-step reaction mechanism in Li-S batteries. The higher reduction peak at 2.30 V corresponds to the reduction of elemental sulfur (S_8_) to long-chain polysulfides Li_2_S_x_ (4 ≤ x ≤ 8), while the lower reduction peak at 2.00 V corresponds to the further reduction of long-chain polysulfides to short-chain sulfides Li_2_S_2_ and Li_2_S. Correspondingly, the oxidation peak at 2.43 V corresponds to the reverse oxidation reaction from short-chain sulfides Li_2_S_2_ and Li_2_S to long-chain polysulfides Li_2_S_x_ (4 ≤ x ≤ 8) and ultimately to elemental sulfur (S_8_). During the initial three cycles, the cyclic voltammetry curves overlap closely, with no significant shifts in the oxidation-reduction peaks, indicating excellent cycle reversibility and stability of Li-S batteries utilizing SiO_2_-g-PAMPSLi@PEO. The constant current charge–discharge profiles at standard temperature (25 °C) and 0.1 C current rate conditions of Li-S batteries employing SiO_2_-g-PAMPSLi@PEO as SPEs (Figure 5B) reveal a continuous charging plateau (~2.20 V) and two discharging plateaus (~2.35 V and ~2.10 V), representing the oxidation reaction from Li_2_S to elemental sulfur S_8_ and the two-step reduction reaction from elemental sulfur S_8_ to polysulfides Li_2_Sx (4 ≤ x ≤ 8) and then to Li_2_S. These steps correspond to the oxidation peak and two reduction peaks observed in the cyclic voltammetry curve, demonstrating that the oxidation-reduction reactions occurring during the charge–discharge cycles of the SiO_2_-g-PAMPSLi@PEO Li-S battery are characteristic of typical electrochemical redox reactions in Li-S batteries. Furthermore, Li-S batteries utilizing SiO_2_-g-PAMPSLi@PEO as SPEs demonstrate outstanding rate capabilities (Figure 5C). As the current rate escalates from 0.1 C to 1 C, the discharge-specific capacities of the Li-S batteries are recorded as 1098.3 mAh g^−1^, 966.3 mAh g^−1^, 777.5 mAh g^−1^, 419.4 mAh g^−1^, and 189.2 mAh g^−1^. Upon reverting back to 0.1 C, the discharge-specific capacity of the batteries can recover to approximately 987.3 mAh g^−1^, underscoring the exceptional reversibility of the Li-S system. Illustrated in Figure 5D are the constant current charge–discharge profiles of Li-S batteries employing SiO_2_-g-PAMPSLi@PEO as SPEs under current rates ranging from 0.1 C to 1 C. The congruence in the shapes of all curves within the graph indicates that the Li-S battery undergoes the same electrochemical reactions at different current rates, devoid of any side reactions.

By employing constant current charge–discharge methodology under standard temperature (25 °C) and at a 0.1 C current rate, the electrochemical performance of Li-S batteries utilizing @PEO as SPEs was assessed, as depicted in Figure 5E, with PEO and SiO_2_@PEO serving as control groups. Upon examination, the initial discharge-specific capacities of the three types of Li-S batteries using PEO, SiO_2_@PEO, and SiO_2_-g-PAMPSLi@PEO as SPEs were recorded as 854.5 mAh g^−1^, 1030.5 mAh g^−1^, and 1211.4 mAh g^−1^, respectively. Following 200 cycles, the discharge-specific capacities of these batteries stood at 395.8 mAh g^−1^, 581.2 mAh g^−1^, and 875.2 mAh g^−1^, respectively. Evidently, the cyclic performance of the Li-S battery employing SiO_2_-g-PAMPSLi@PEO surpassed that of the other two variants, maintaining a Coulombic efficiency of approximately 100%. This enhancement can be attributed to the superior lithium-ion conductivity of SiO_2_-g-PAMPSLi@PEO, facilitating more thorough electrochemical reactions. Additionally, the favorable compatibility between SiO_2_-g-PAMPSLi and the polymer PEO enhances the contact performance between the composite solid electrolyte and the electrodes, optimizing discharge capacity.

## 4. Conclusions

To mitigate the inert nature of SiO_2_ Nps, their tendency for aggregation, and their inadequate inorganic–organic compatibility with the polymer PEO, we employed an ARGET-ATRP approach involving the grafting of sulfonic acid-functionalized organic polymer chains onto SiO_2_ Nps. This strategic modification was pursued to confer Li^+^ conductivity characteristics to SiO_2_ Nps and improve their compatibility with PEO. Subsequently, the modified nano fillers were blended with PEO using a solution-based method to fabricate SPE films. This approach ultimately led to the development of a polymer composite solid electrolyte. Experimental results demonstrate that this electrolyte manifests reduced crystallinity and exceptional Li^+^ conductivity, thereby displaying remarkable electrochemical performance during charge–discharge cycling in solid-state Li-S batteries.

## Data Availability

Data are included in the article and Supplementary Materials. The data that support the findings of this study are available from the corresponding author, [W.R.], upon reasonable request.

## Supplementary Materials

The following supporting information can be downloaded at: https://www.mdpi.com/article/10.3390/polym16081128/s1, Figure S1. The 1H NMR spectrum of pre-initiator. Figure S2. SEM images of (A) SiO_2_ Nps and (B) SiO_2_ Nps initiator. Figure S3. SEM images of SPEs with varying levels of (A–D) SiO_2_ and (E–I) SiO_2_-g-PAMPSLi content. Figure S4. The stress–strain curves of different SPEs. Figure S5. The stress–strain curves of SPEs with varying levels of SiO_2_-g-PAMPSLi content. Table S1. DSC results of SPEs containing varying amounts of SiO_2_. Table S2. DSC results of SPEs containing varying amounts of SiO_2_-g-PAMPSLi. Figure S6. The chronoamperometric curves at room temperature (25 °C) and the corresponding pre- and post-polarization AC impedance spectra of the SPEs with varying levels of SiO_2_-g-PAMPSLi content. Table S3. Li^+^ migration number of SPEs containing varying amounts of SiO_2_-g-PAMPSLi. Table S4. The electrochemical performances of lithium–sulfur batteries with PEO-based SPEs. References [50,51,52,53,54,55,56,57,58] are cited in the supplementary materials.
