# Supplementary material for: ARGET-ATRP-Mediated Grafting of Bifunctional Polymers onto Silica Nanoparticles Fillers for Boosting the Performance of High-Capacity All-Solid-State Lithium–Sulfur Batteries with Polymer Solid Electrolytes
Source: Polymers (Basel). 2024 Apr 17;16(8):1128. doi: 10.3390/polym16081128 (PMC11054452; doi:10.3390/polym16081128)
Supplement: Supplementary file 1 [file polymers-16-01128-s001.zip › polymers-2925490-supplementary.pdf]

polymers

MDPI

Supplementary Materials

ARGET-ATRP Mediated Grafting of Bifunctional Polymers onto Silica Nanoparticles Fillers for Boosting the Performance of High-Capacity All-Solid-State Lithium-sulfur Batteries with Polymer Solid Electrolytes

Liang Wang [1], Junyue Huang [1], Yujian Shen [1], Mengqi Ma [1], Wenhong Ruan [1,2]* and Mingqiu Zhang [1,2]

1 Key Laboratory for Polymeric Composite and Functional Materials of Ministry of Education, GD HPPC Lab, School of Chemistry, Sun Yat-sen University, Guangzhou 510275, China; wangln57@mail2.sysu.edu.cn (L.W.); huangjy329@mail2.sysu.edu.cn (J.H.); shenyj7@mail2.sysu.edu.cn (Y.S.); mamq6@mail2.sysu.edu.cn (M.M.); ceszmq@mail.sysu.edu.cn (M.Z.)

2 Guangdong Provincial Laboratory of Chemistry and Fine Chemical Engineering Jieyang Center, Jieyang 515200, China

* Correspondence: cesrwh@mail.sysu.edu.cn; Tel.: +86-020-84114008

Synthesis of SiO_2 Nps

Synthesis of SiO_2 NPs was conducted using a modified Stöber method. [1] The specific procedure is as follows: Initially, 4.5 mL of TEOS was dissolved in 30 mL of anhydrous ethanol to prepare solution A. Subsequently, a mixture of 7 mL of concentrated ammonia solution, 8 mL of deionized water, and 50 mL of anhydrous ethanol was prepared at 50°C under stirring at 500 rpm, designated as solution B. With continuous stirring, solution A was slowly added dropwise into solution B, and the reaction was carried out at 50°C for 4 hours, followed by an additional 20 hours at room temperature. After completion of the reaction, the solid product was separated by centrifugation at 8000 rpm and washed three times with anhydrous ethanol, followed by drying.

Activation of Nano SiO_2

(1) Add 9mL of hydrogen peroxide solution to a round bottom flask and slowly add 21mL of concentrated sulfuric acid in an ice water bath, stirring for 30 minutes; (2) Add 1g of nano SiO_2 powder and sonicate for 15 minutes; (3) React at 90°C for 24 hours; (4) Using centrifugal separation, wash with deionized water until the clear liquid is neutral, and dry in a vacuum oven at 70°C for 24 hours before use.

Synthesis of the pre-initiator

To synthesize the pre-initiator, 2.96 mL of 2-bromo-2-methylpropanoyl bromide was dissolved in 30 mL of anhydrous toluene, yielding solution C. In a separate flask, 4.66 mL of 3-aminopropyltriethoxysilane and 3.34 mL of triethylamine were dissolved in 70 mL of anhydrous toluene. This mixture, designated as solution D, was prepared under stirring at 500 rpm in an ice bath and under an argon atmosphere for 15 minutes. Subsequently, while maintaining the ice bath and stirring, solution C was slowly added dropwise into solution D. The reaction proceeded for 4 hours, followed by an additional 20 hours at room temperature. After completion of the reaction, insoluble solids were removed by vacuum filtration. The filtrate was subjected to solvent removal using a rotary evaporator, resulting in the formation of a pale yellow oily liquid as the final product.

Citation: To be added by editorial staff during production.

Academic Editor: Firstname Lastname

Received: date
Revised: date
Accepted: date
Published: date

Copyright: © 2024 by the authors. Submitted for possible open access publication under the terms and conditions of the Creative Commons Attribution (CC BY) license (https://creativecommons.org/licenses/by/4.0/).

Activation of SiO_2 Nps

To activate the SiO_2 Nps, a solution of 9 mL of hydrogen peroxide was slowly added dropwise to 21 mL of concentrated sulfuric acid under stirring at 500 rpm and in an ice bath. The resulting solution, designated as solution E, was stirred for 30 minutes. Subsequently, 1 g of previously synthesized SiO_2 nanoparticles was added to solution E, followed by ultrasonic dispersion for 15 minutes. The mixture was then subjected to a reaction at 90°C for 24 hours. The resulting solid was centrifuged at 8000 rpm, and the solid was washed with deionized water until the supernatant reached a neutral pH. The activated SiO_2 nanoparticles were then dried.

Synthesis of SiO_2 Nps initiator

To synthesize the SiO_2 nanoparticle initiator, 0.8 g of the previously prepared pre-initiator was dissolved in 10 mL of anhydrous toluene, yielding solution F. Subsequently, 1 g of activated SiO_2 nanoparticles and 40 mL of anhydrous toluene were added to a Schlenk flask, followed by ultrasonic dispersion for 15 minutes to obtain solution G. Solution F was then slowly added dropwise to solution G. The resulting mixture underwent three freeze-pump-thaw cycles using liquid nitrogen and Ar, followed by stirring at 500 rpm at room temperature for 2 days. The solid product was separated by centrifugation at 8000 rpm and washed twice with anhydrous toluene, anhydrous acetone, and anhydrous ethanol, respectively. After vacuum drying, the SiO_2 nanoparticle initiator was obtained.

Synthesis of SiO_2-g-PAMPSLi

The polymerization reaction was conducted using ARGET-ATRP with the following specific steps: Initially, 250 mL of water and 250 mL of anhydrous methanol were added to a 250 mL flask, followed by the addition of 103.6 g of AMPS. The mixture was stirred at 500 rpm for 15 minutes. While maintaining stirring, 21 g of lithium hydroxide monohydrate was added to the solution, and after 30 minutes, 4 g of the previously synthesized SiO_2 nanoparticle initiator was added, followed by ultrasonic dispersion for 20 minutes. The reaction system was then purged with argon for 15 minutes. Under a continuous flow of argon, 0.67 g of copper bromide, 1.36 mL of HMTETA, and 0.88 g of ascorbic acid were added sequentially. The reaction was carried out at 40°C under an argon atmosphere for 36 hours. After the reaction, the solid product was separated by centrifugation at 8000 rpm and washed three times with deionized water. The SiO_2-g-PAMPSLi was obtained after vacuum drying.

Material Characterizations

The synthesized pre-initiator was performed by dissolving it in deuterated chloroform and analyzing its chemical structure using nuclear magnetic resonance (NMR, Varian INOVA500NB, Japan). The morphological structure and elemental mapping of SiO_2-g-PAMPSLi were observed by scanning electron microscopy (SEM, Hitachi S-4800, Japan) and transmission electron microscopy (TEM, JEOL JEM-2010HR, Japan). Elemental mapping was observed by energy dispersive X-ray spectroscopy (EDS, Hitachi S-4800, Japan). Fourier Transform Infrared Spectroscopy (FTIR Tensor27, Germany) was carried out to characterize the molecular structure. The decomposition of the grafted polymer on the surface of SiO_2 Nps was measured by the thermogravimetric analysis (TGA, TA Instrument TGA-Q50, America). The crystal structure of individual SiO_2 and SiO_2-g-PAMPSLi composite were measured by X-ray diffraction (XRD, Bruker D8 ADVANCE, Germany) from 5º to 60º with a scanning rate of 6º min-1 at room temperature. The influence of nanoparticles on the crystallization behavior of PEO was investigated by XRD and differential scanning calorimetry (DSC, Waters DSC-Q10, China) characterization. The Li-ion conductivity (s) of SiO_2-g-PAMPSLi@PEO QSSCE was measured by the electrochemical impedance spectroscopy (EIS) method in an electrochemical workstation (CHI760E, CH Instrument, Shanghai, China) with a voltage amplitude of 5 mV at the frequency range of 0.1 Hz to 1 MHz.

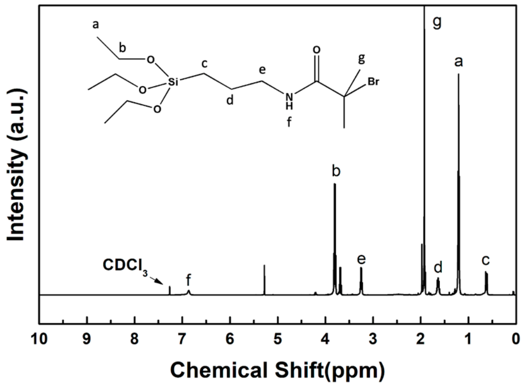

Figure S1. The 1H NMR spectrum of pre-initiator

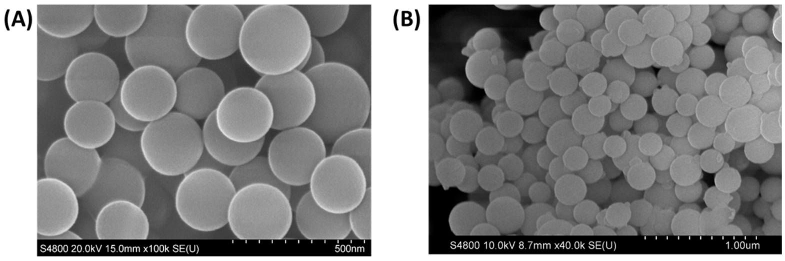

Figure S2. SEM images of (A) SiO_2 Nps and (B) SiO_2 Nps initiator.

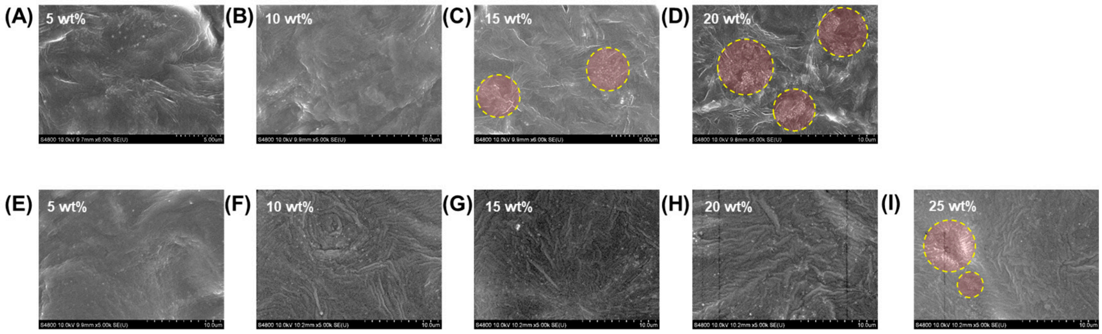

Figure S3. SEM images of SPEs with varying levels of (A)- (D) SiO_2 and (E) - (I) SiO_2-g-PAMPSLi content.

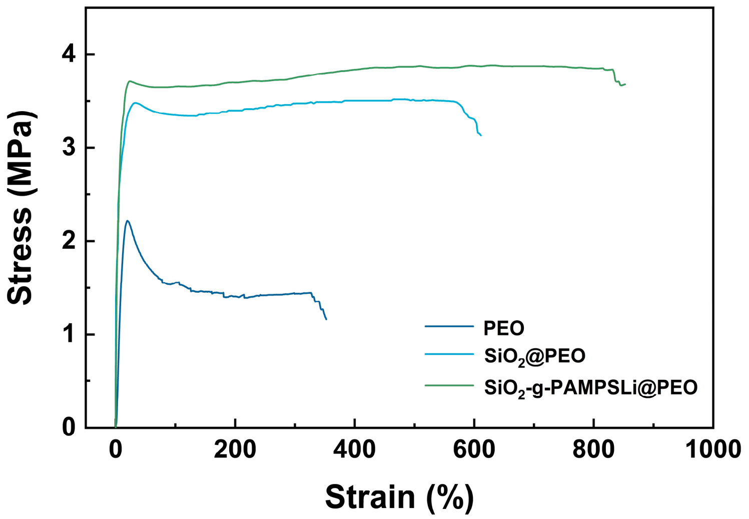

Figure S4. The stress-strain curves of different SPEs.

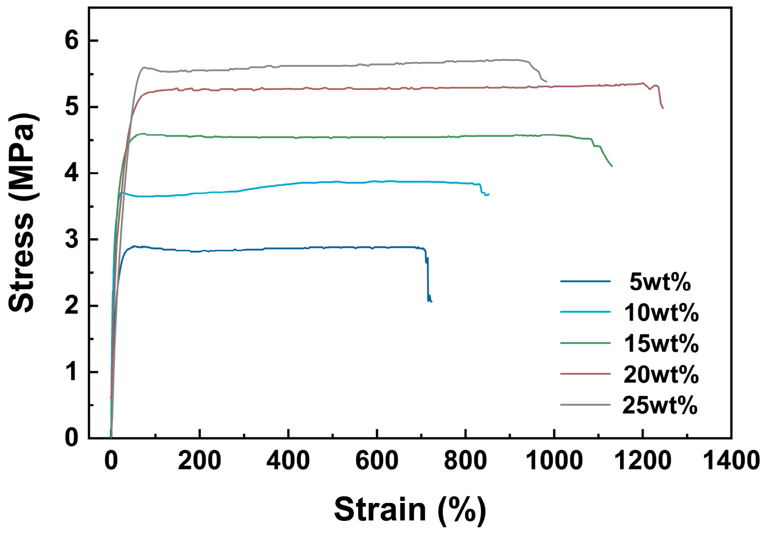

Figure S5. The stress-strain curves of SPEs with varying levels of SiO_2-g-PAMPSLi content.

Table S1. DSC results of SPEs containing varying amounts of SiO_2.

SiO_2 content (wt%)	T_m (°C)	ΔH_m (J g^{-1})	χ_c (%)
5	52.3	63.8	44
10	51.2	54.2	39
15	50.5	53.1	40
20	50.4	53.2	42

Table S2. DSC results of SPEs containing varying amounts of SiO_2-g-PAMPSLi.

SiO_2-g-PAMPSLi content (wt%)	T_m (°C)	**Δ**H_m (J g^{-1})	**χ**$_c$ (%)
5	51.5	60.0	40
10	50.2	50.0	36
15	49.3	43.8	33
20	49.0	30.4	24
25	49.1	34.8	29

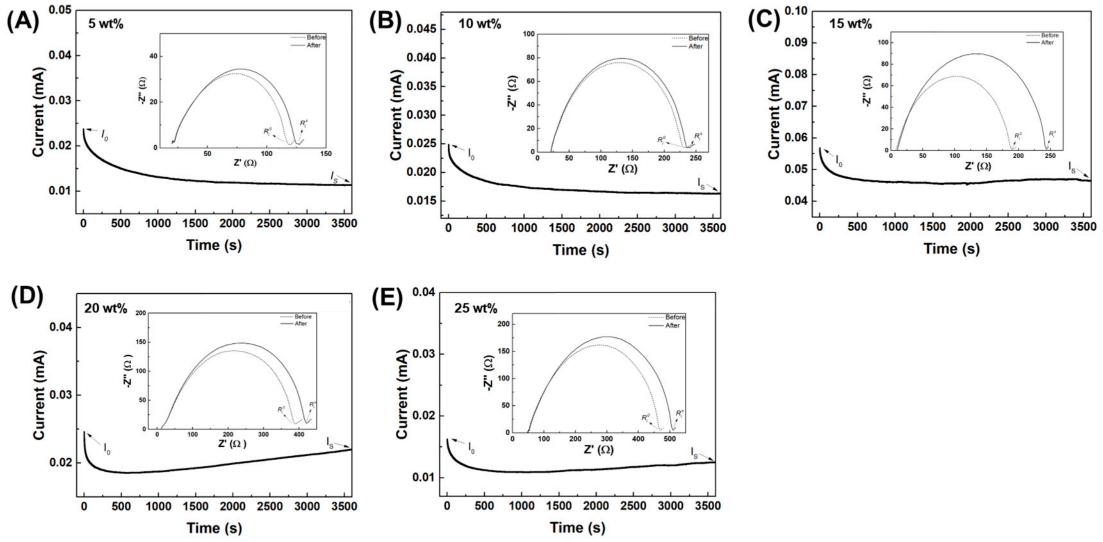

Figure S6. The chronoamperometric curves at room temperature (25°C) and the corresponding pre- and post-polarization AC impedance spectra of the SPEs with varying levels of SiO_2-g-PAMPSLi content.

Table S3. Li+ migration number of SPEs containing varying amounts of SiO_2-g-PAMPSLi

SiO_2-g-PAMPSLi content (wt%)	I_0（mA）	I_S（mA）	R_0（**Ω**）	R_S（**Ω**）	t^+
5	0.0237	0.0114	120	127	0.40
10	0.0249	0.0164	235	240	0.45
15	0.0568	0.0465	190	244	0.48
20	0.0246	0.0220	390	421	0.49
25	0.0162	0.0126	469	510	0.51

Table S4. The electrochemical performances of lithium–sulfur batteries with PEO–based SPEs.

Composite solid–stateelec-trolytes	Temperature(℃)	Current density	Cycle number	Capacity (mAh g^{-1})	Retention rate (%)	Ref.
PEO/LiFTFSI	60	0.1 C	60	800	53.0	[2]

PEO/PTFE/LiTFSI	55	0.03 C	60	630	77.0	[3]
PEO/LiTFSI/PIM	60	0.5 C	100	730	61.8	[4]
PI@PEO/LiTFSI	80	0.8 C	150	333.2	68.0	[5]
PEO/LiTFSI/LATP	75	0.1 C	120	784	43.7	[6]
PEO/LiTFSI/LGPS	60	0.1 C	50	588	33.2	[7]
PEO/LiClO4/$Li_7P_3S_{11}$	25	0.05 C	60	394	47.7	[8]
PEO/LiTFSI/ALF	60	0.05 C	100	907.6	73.5	[9]
PEO/LiTFSI/LLZTO	60	0.2 C	50	633	68.1	[10]
PEO/LiTFSI/TiO2/Al_2O_3	60	0.1 C	120	640	45.2	[11]
SiO_2-g-PAMPSLi@PEO	25	0.1C	200	875.2	72.2	This work

References

[1] W. Stöber, A. Fink, E. Bohn, Controlled growth of monodisperse silica spheres in the micron size range, *Journal of Colloid and Interface Science* **1968,** *26,* 62-69. https://doi.org/10.1016/0021-9797(68)90272-5

[2] G.G. Eshetu, X. Judez, C. Li, M. Martinez-Ibanez, I. Gracia, O. Bondarchuk, J. Carrasco, L.M. Rodriguez-Martinez, H. Zhang, M. Armand, Ultrahigh Performance All Solid-State Lithium Sulfur Batteries: Salt Anion's Chemistry-Induced Anomalous Synergistic Effect, *J Am Chem Soc* **2018,** *140,* 9921-9933. 10.1021/jacs.8b04612

[3] R. Fang, H. Xu, B. Xu, X. Li, Y. Li, J.B. Goodenough, Reaction Mechanism Optimization of Solid - State Li - S Batteries with a PEO - Based Electrolyte, *Advanced Functional Materials* **2020,** *31,* 10.1002/adfm.202001812

[4] Y. Ji, K. Yang, M. Liu, S. Chen, X. Liu, B. Yang, Z. Wang, W. Huang, Z. Song, S. Xue, Y. Fu, L. Yang, T.S. Miller, F. Pan, PIM - 1 as a Multifunctional Framework to Enable High - Performance Solid - State Lithium - Sulfur Batteries, *Advanced Functional Materials* **2021,** *31,* 10.1002/adfm.202104830

[5] X. Gao, X. Zheng, J. Wang, Z. Zhang, X. Xiao, J. Wan, Y. Ye, L.Y. Chou, H.K. Lee, J. Wang, R.A. Vila, Y. Yang, P. Zhang, L.W. Wang, Y. Cui, Incorporating the Nanoscale Encapsulation Concept from Liquid Electrolytes into Solid-State Lithium-Sulfur Batteries, *Nano Lett* **2020,** *20,* 5496-5503. 10.1021/acs.nanolett.0c02033

[6] S. Hong, Y. Wang, N. Kim, S.B. Lee, Polymer-based electrolytes for all-solid-state lithium–sulfur batteries: from fundamental research to performance improvement, *Journal of Materials Science* **2021,** *56,* 8358-8382. 10.1007/s10853-021-05832-2

[7] M. Li, J.E. Frerichs, M. Kolek, W. Sun, D. Zhou, C.J. Huang, B.J. Hwang, M.R. Hansen, M. Winter, P. Bieker, Solid - State Lithium - Sulfur Battery Enabled by Thio - LiSICON/Polymer Composite Electrolyte and Sulfurized Polyacrylonitrile Cathode, *Advanced Functional Materials* **2020,** *30,* 10.1002/adfm.201910123

[8] X. Xu, G. Hou, X. Nie, Q. Ai, Y. Liu, J. Feng, L. Zhang, P. Si, S. Guo, L. Ci, Li7P3S11/poly(ethylene oxide) hybrid solid electrolytes with excellent interfacial compatibility for all-solid-state batteries, *Journal of Power Sources* **2018,** *400,* 212-217. 10.1016/j.jpowsour.2018.08.016

[9] W. Kou, J. Wang, W. Li, R. Lv, N. Peng, W. Wu, J. Wang, Asymmetry-structure electrolyte with rapid Li+ transfer pathway towards high-performance all-solid-state lithium–sulfur battery, *Journal of Membrane Science* **2021,** *634,* 10.1016/j.memsci.2021.119432

[10] Y. Liu, H. Liu, Y. Lin, Y. Zhao, H. Yuan, Y. Su, J. Zhang, S. Ren, H. Fan, Y. Zhang, Mechanistic Investigation of Polymer‐Based All‐Solid‐State Lithium/Sulfur Battery, *Advanced Functional Materials* **2021,** *31,* 10.1002/adfm.202104863

[11] T. Babkova, R. Kiefer, Q.B. Le, Hybrid Electrolyte Based on PEO and Ionic Liquid with In Situ Produced and Dispersed Silica for Sustainable Solid-State Battery, *Sustainability* **2024,** *16,* 1683-1696. https://doi.org/10.3390/su16041683
